# Kidney disease and transplantation in childhood cancer survivors

**DOI:** 10.1007/s00467-025-06985-x

**Published:** 2025-10-20

**Authors:** Abigail S. Kane, Wendy C. Bravo

**Affiliations:** 1 Department of Pediatrics, Children’s Hospital Los Angeles, Los Angeles, CA USA; 2 Keck School of Medicine of University of Southern California, Los Angeles, CA USA; 3 Division of Pediatric Nephrology, Children’s Hospital Los Angeles, Los Angeles, CA USA

**Keywords:** Childhood cancer, Chronic kidney disease, Kidney failure, Kidney transplant, Cancer screening

## Abstract

Advancements in pediatric cancer treatment protocols have significantly improved long-term survival. This has been accompanied by a growing recognition of morbidity and mortality associated with late effects of treatment, including kidney disease. Surviving cancer in childhood implies exposure to multiple nephrotoxic insults, some of which carry a greater risk for the development of chronic kidney disease and progression to kidney failure than others. In childhood cancer survivors who develop kidney failure, a kidney transplant is a potential life-prolonging treatment option. However, the optimal timing of transplant in relation to remission, particularly when weighing morbidity associated with increased time on dialysis against the risk of cancer recurrence associated with transplant immunosuppression, remains controversial. This review explores nephrotoxicity and kidney-related complications of cancer treatment, kidney disease screening guidelines, and considerations for kidney transplant in childhood cancer survivors including timing from remission to transplant, cancer recurrence in the setting of post-transplant immunosuppression, and transplant outcomes.

**Graphical Abstract:**

**Figure:**
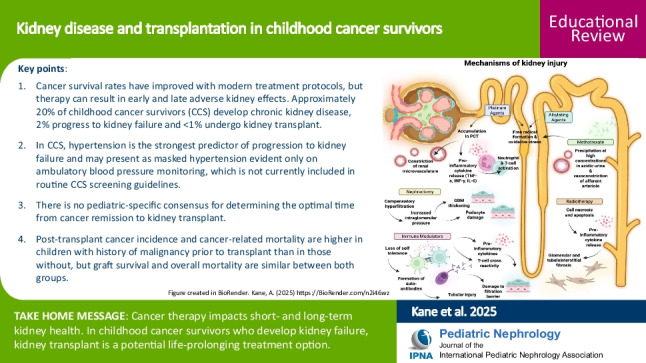
A higher resolution version of the Graphical abstract is available as Supplementary information

**Supplementary Information:**

The online version contains supplementary material available at 10.1007/s00467-025-06985-x.

## Introduction

**Fig. 1:**
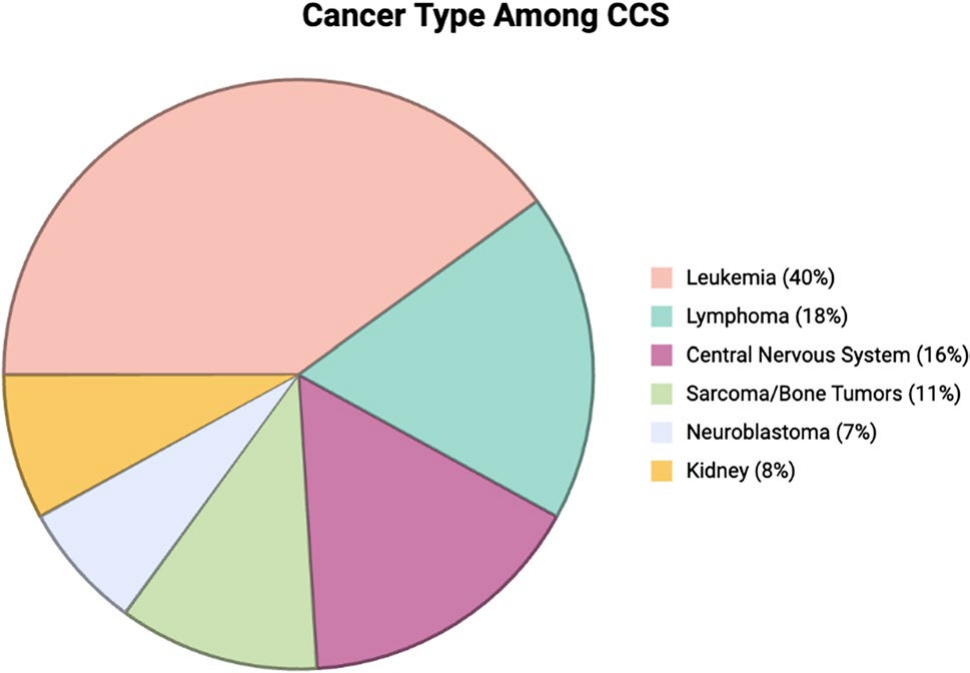
Cancer epidemiology. This figure displays the most frequent pediatric cancers among survivors. Data was obtained from 34,230 5-year survivors in the Childhood Cancer Survivor Study diagnosed before 21 years of age between 1970 and 1999 [3]. Created in BioRender. Kane A (2025) https://BioRender.com/pjgxen6

Cancer is the second leading cause of death among children in the USA aged 1–14 years and a major contributor to mortality in children and adolescents worldwide [1, 2]. While survival rates are largely affected by cancer type (Fig. 1), an upsurge in the development of clinical trials has led to more effective treatment protocols and a notable decline in childhood cancer mortality. Five-year survival rates now reach 85%, a remarkable improvement from 58% reported in the 1970 s [1]. However, surviving cancer in childhood is associated with increased long-term morbidity and mortality. Over 70% of survivors develop more than two chronic health conditions, and late survivors (> 40 years from diagnosis) are at increased risk of dying from common causes of mortality, including a 6.9 times higher mortality rate from kidney failure, compared to the general population [3, 4]. Cumulative nephrotoxic effects of cancer and its treatment leave survivors vulnerable to developing chronic kidney disease and requiring kidney replacement therapy. This review therefore focuses on the causes and implications of kidney disease and considerations for kidney transplant in childhood cancer survivors and is intended for providers of pediatric nephrology care.

## Long-term adverse effects of cancer therapy on the kidney

**Fig. 2:**
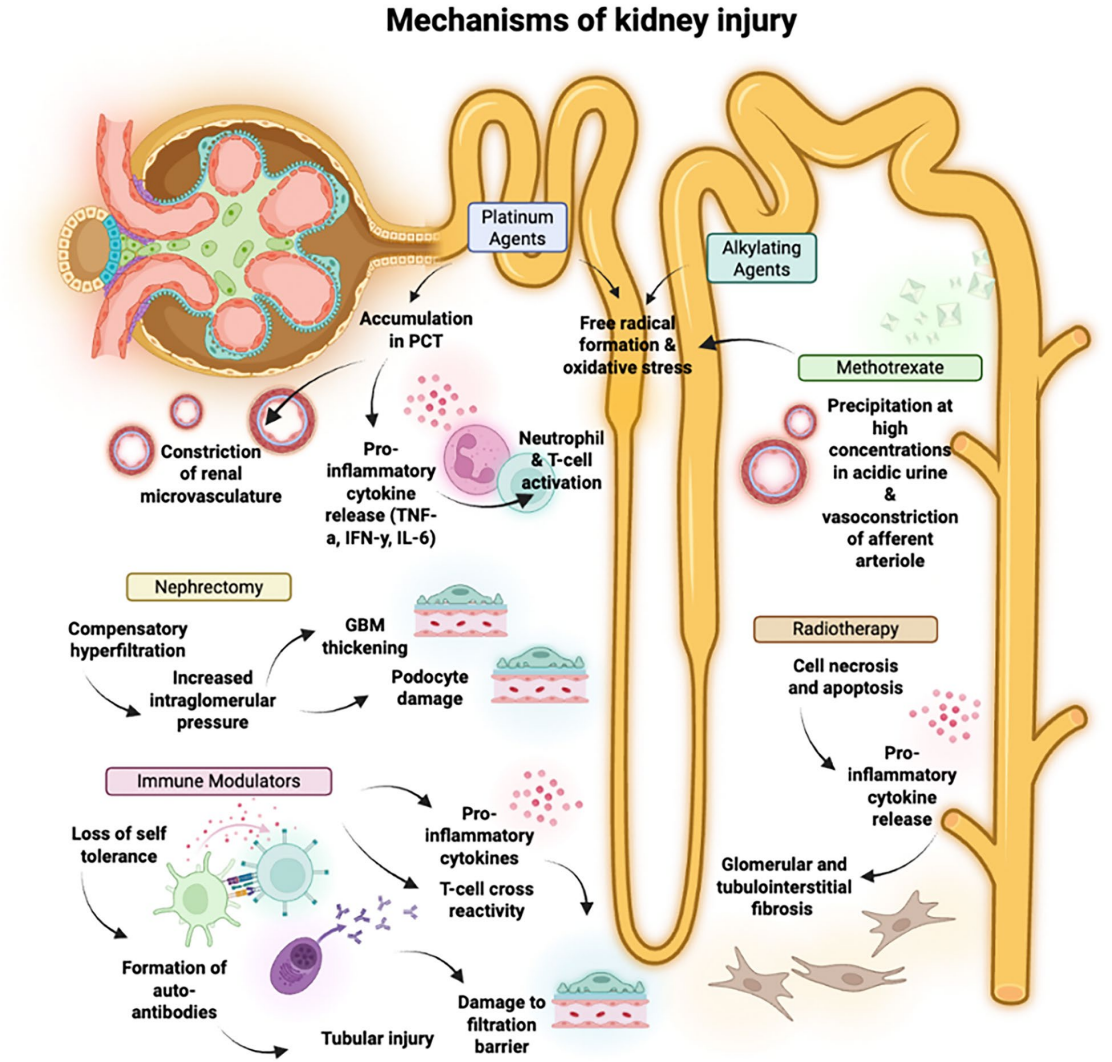
Mechanisms of kidney injury in cancer therapy, Abbreviations: PCT, proximal convoluted tubule; TNF-α, tumor necrosis factor alpha; IFN-γ, interferon gamma; IL-6, interleukin-6; GBM, glomerular basement membrane. Created in BioRender. Kane A (2025) https://BioRender.com/n2i46wz

The etiology of kidney disease in childhood cancer survivors (CCS) is multifactorial. Renal infiltration, repeated acute kidney injury (AKI), and the nephrotoxic effects of cancer therapy (i.e., chemotherapy, radiotherapy, targeted immunotherapy, hematopoietic stem cell transplantation [HSCT], and nephrectomy) (Fig. 2) all contribute to CCS having a nearly fivefold risk of developing chronic kidney disease (CKD) compared to their siblings without a history of cancer [5]. Broadly, children receiving higher doses of chemotherapy and/or radiotherapy, coadministration of nephrotoxic agents, and those with decreased kidney reserve due to prior nephrectomy or kidney injury are at greater risk of both acute and chronic adverse kidney effects. While the mechanisms leading to kidney injury vary, the most common long-term sequelae of cancer therapy include proteinuria, hypertension, persistent electrolyte derangements, and CKD, with 15% of those with CKD progressing to kidney failure [5, 6]**.**

### Chemotherapy

Patients treated with chemotherapy regimens that include cisplatin, alkylating agents (i.e., ifosfamide, cyclophosphamide), and high-dose methotrexate more frequently develop acute and late adverse kidney effects [7, 8]. Cisplatin induces apoptosis of cancer cells by crosslinking DNA, which distorts DNA structure and leads to irreversible cell damage. The drug accumulates in cells of the S3 segment of the proximal tubule and causes toxicity by inducing a pro-inflammatory cytokine response (i.e., TNF-α, IL-6, IFN-gamma) that promotes neutrophil and T-cell-mediated inflammation [9]. Additionally, it causes oxidative stress and vasoconstriction of the renal microvasculature [9, 10]. Altogether, these effects result in AKI that is generally reversible and non-oliguric and acute tubular dysfunction that progresses to chronic tubulopathy in 10% of cases, characterized by salt wasting, hypomagnesemia, hypophosphatemia, tubular proteinuria, or overt Fanconi’s syndrome [11, 12]. Acute and late kidney involvement affects up to 45% of patients who receive cisplatin [13].

Cyclophosphamide and its more nephrotoxic synthetic structural isomer, ifosfamide, are alkylating agents that result in cell death through inhibition of DNA synthesis. While these mechanistic effects generally impact rapidly dividing cells, toxicity to tubular cells mainly occurs due to oxidative stress induced by the drugs’ active metabolites. Similar to cisplatin-induced tubular dysfunction, alkylating agents can result in tubulopathies of variable severity and chronicity, affecting up to 30% of children and persisting in up to two-thirds of these patients into adulthood [14]. Manifestations of toxicity aside from hemorrhagic cystitis and syndrome of inappropriate antidiuretic hormone secretion (SIADH) include proximal or distal renal tubular acidosis, tubular proteinuria, and Fanconi syndrome with hypophosphatemia that can be severe and persistent enough to result in rickets [14]**.**

Methotrexate is an antimetabolite that halts DNA synthesis by competitively inhibiting dihydrofolate reductase. When administered at high doses (≥ 500 mg/m^2^), as often indicated for the management of acute lymphoblastic leukemia, osteosarcoma, lymphoma, and other cancers, methotrexate can acutely induce afferent arteriolar vasoconstriction and precipitate in the tubular lumen. This results in free radical formation and subsequent tubular cell injury, necrosis, and reversible non-oliguric AKI in 2–12% of patients [11, 15]. Notably, a reduced glomerular filtration rate (GFR) in the setting of methotrexate-induced AKI or preexisting kidney injury results in delayed drug clearance, higher serum methotrexate levels, and further toxicity including severe mucositis, myelosuppression, hepatotoxicity, and neurotoxicity [15]. The acute adverse effects of methotrexate can be somewhat mitigated by interventions such as hyperhydration, leucovorin (folinic acid), and glucarpidase (Table 1) [16–18].

**Table 1:** Cancer therapy-specific strategies for preventing kidney injury

Cancer therapy	Mechanism of kidney injury	Kidney injury prevention strategies	Mechanism of injury prevention
Cisplatin	Pro-inflammatory cytokine release, PMN and T-cell recruitment Oxidative stress Vasoconstriction of renal microvasculature	Hyperhydration ± furosemide	Improves renal clearance of drug, prevents drug accumulation that leads to inflammatory response
		Sodium chloride (0.9% NaCl)	Exact mechanism is unknown. Change in osmolarity may trigger cellular stress response that modulates sensitivity to cisplatin and limits nephrotoxicity [80]. Higher IC chloride levels may prevent cisplatin hydrolysis and minimize oxidative stress caused by toxic metabolites [81]
		Magnesium sulfate	Likely reduces tubular cisplatin accumulation, oxidative stress and tubular injury
		Acetazolamide	Alkalinizes urine which improves cisplatin metabolite clearance. Anti-inflammatory effect through inhibition of prostaglandin synthesis. Antioxidant properties reduce oxidative stress [82]
Cyclophosphamide/ Ifosfamide	Oxidative stress	Hyperhydration, frequent bladder emptying	Improves renal clearance of drug metabolites and minimizes contact time with urothelium
		Mesna	Binds acrolein (urotoxic metabolite) to prevent hemorrhagic cystitis and secondary obstructive uropathy
High-dose methotrexate	Afferent arteriolar vasoconstriction Tubular precipitation causes free radical formation and oxidative stress	Hyperhydration	Improves renal clearance of drug, prevents precipitation in renal tubules
		Leucovorin (folinic acid)* *Folate analogue*	Restores pool of folate metabolites used for DNA synthesis to reduce systemic effects of HDMTX
		Glucarpidase *Recombinant zinc-dependent metallopeptidase*	Cleaves methotrexate into non-toxic metabolites. FDA approved single-dose rescue for children with serum MTX level ≥ 1 mmol/L. Reduces MTX levels by up to 99% within an hour
Radiotherapy	Oxidative stress RAAS activation	Limit total RT dose < *10–15 Gy*	Limits exposure
Nephrectomy *Complete or partial*	Compensatory hyperfiltration of remaining nephrons, podocyte damage and GBM thickening	Patient education *Counseling on single kidney health risks and avoidance of nephrotoxic drugs (i.e., NSAIDS)*	Reduces incidence of future episodes of AKI and delays onset of CKD

Some commonly used therapy-specific strategies for prevention of kidney injury. Adjustment of drug dosing for kidney impairment, avoidance of concurrent and future exposure to other nephrotoxic drugs, and limiting total doses of chemotherapeutic agents and radiotherapy are of paramount importance. *Leucovorin is not used to prevent methotrexate-induced kidney injury. It mitigates the systemic effects of HDMTX in rapidly dividing cells, which can be more severe in the setting of reduced kidney drug clearance *AKI*, acute kidney injury, *CKD* chronic kidney disease, *FDA* Food and Drug Administration, *IC* intracellular, *HDMTX* high-dose methotrexate, *MTX* methotrexate, *PMN* polymorphonuclear leukocytes, *RAAS* renin–angiotensin–aldosterone system, *RT* radiotherapy, *GBM* glomerular basement membrane

In contrast to its acute effects, the long-term kidney implications of methotrexate are not as well characterized. Some cohorts have found late effects of both glomerular and tubular dysfunction in survivors who received high-dose methotrexate, evidenced by reduced GFR with elevated cystatin C and elevated urine neutrophil gelatinase-associated lipocalin, respectively [7]. Conversely, others have not found a statistically significant reduction in GFR over time in adults treated with high-dose methotrexate during childhood [19].

### Novel immunotherapies

**Fig. 3:**
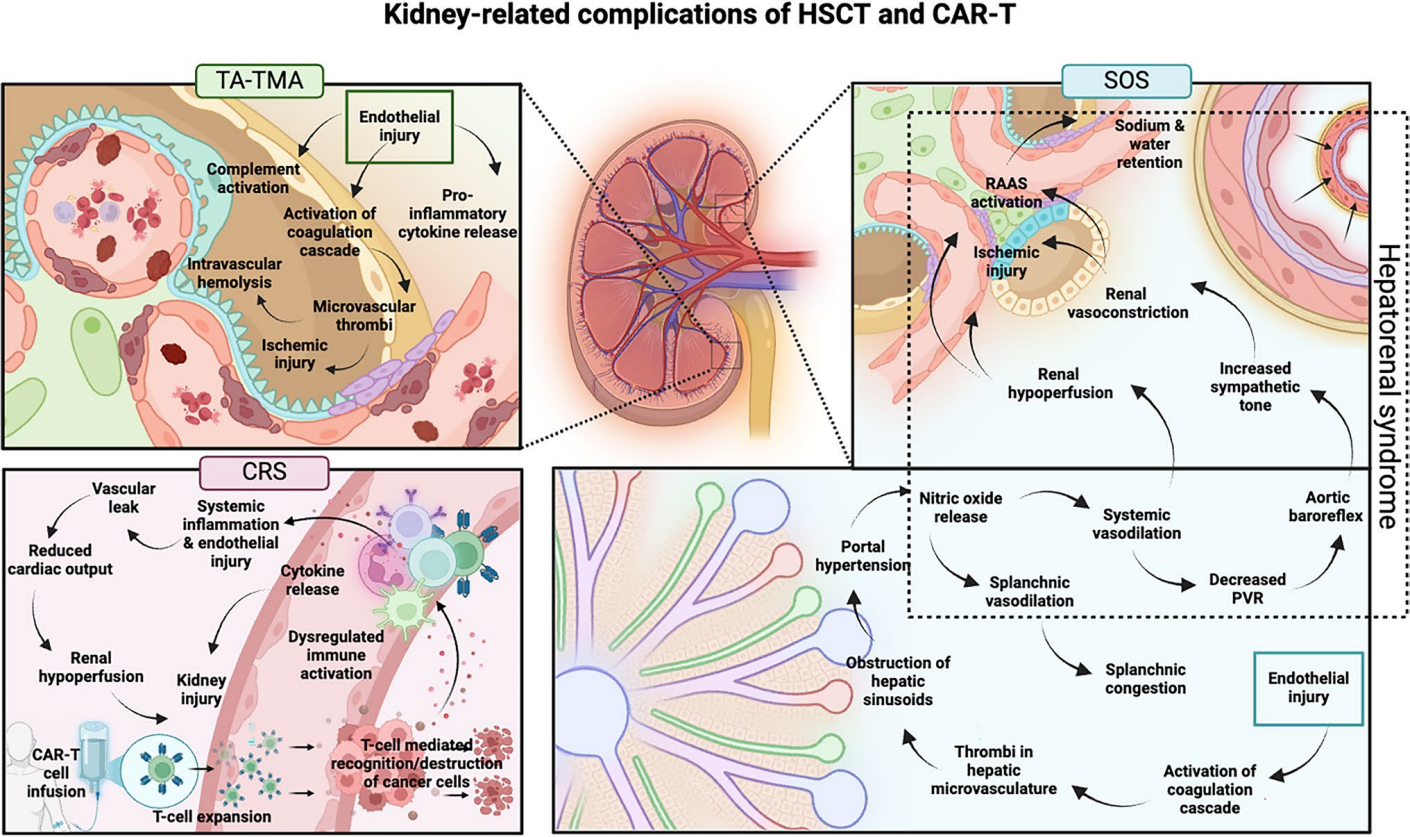
Kidney-related complications of HSCT and CAR-T. Abbreviations: TA-TMA, transplant-associated thrombotic microangiopathy; CRS, cytokine release syndrome; CAR-T, chimeric antigen receptor T-cell; PVR, peripheral vascular resistance; RAAS, renin–angiotensin–aldosterone system; SOS, sinusoidal obstruction syndrome. Created in BioRender. Kane A (2025) https://BioRender.com/f92qw6h

In recent years, cancer treatment has undergone a revolutionary shift with a growing focus on immunotherapy, allowing for improved cancer outcomes and reduced reliance on traditional cytotoxic chemotherapy. However, targeted immunotherapies such as immune checkpoint inhibitors (i.e., pembrolizumab), cytokine therapy (interferon-a, high-dose IL-2), and bispecific monoclonal antibodies (i.e., blinatumomab) can result in kidney complications including acute interstitial nephritis, nephrotic syndrome, glomerulonephritis, tubulopathies with electrolyte disturbances, thrombotic microangiopathy (TMA), and AKI, among others [20, 21]. Other novel cancer treatments, including chimeric antigen receptor T-cell (CAR-T) therapy, can also contribute to kidney injury. CAR-T therapy allows for autologous T-cell-mediated recognition and destruction of cancer cells. However, T-cell receptor binding with tumor-specific surface antigens can trigger unchecked activation of immune cells (i.e., CAR-T-cells, T-cells, B-cells, macrophages, dendritic cells) and subsequent release of pro-inflammatory cytokines, which results in a vast spectrum of systemic inflammation termed cytokine release syndrome (CRS) (Fig. 3) [22]. Patients with severe CRS experience multi-organ damage, including kidney injury [23]. This is mediated by direct cytokine toxicity and renal hypoperfusion, the latter of which occurs because of reduced cardiac output, endothelial dysfunction, and vascular leak in the setting of systemic inflammation. Kidney injury manifests with electrolyte derangements and acidosis and can progress to kidney failure requiring dialysis [22, 24, 25].

### Radiotherapy

Radiotherapy induces acute damage through direct ionization of DNA, production of reactive oxygen species resulting in oxidative stress, and activation of the renin–angiotensin–aldosterone system [24]. Radiotherapy results in structural damage to DNA molecules and subsequent necrosis and apoptosis. Surviving cells remain senescent, producing pro-inflammatory cytokines that contribute to both glomerular and tubulointerstitial fibrosis that characterize radiation nephropathy. Acutely, radiation nephropathy may be subclinical, with proteinuria and/or azotemia detectable before the onset of fatigue, edema, anemia, and hypertension [26]. Chronic radiation nephropathy generally ensues 12–18 months after therapy and is characterized by hypertension, proteinuria, and CKD secondary to renal scarring and atrophy [27]. The risk of developing radiation nephropathy is highest with total radiotherapy doses greater than 15 Gy [5]. With adjuvant chemotherapy protocols, children now generally receive lower doses of radiotherapy, making radiation nephropathy significantly less common than in previous decades. However, children undergoing HSCT receive total body irradiation prior to transplant and are therefore among the children at higher risk for late effects of radiotherapy.

### Nephrectomy

Unilateral nephrectomy results in compensatory hyperfiltration of the remaining glomeruli, which initially helps maintain kidney function. However, increased intraglomerular pressure leads to podocyte damage and glomerular basement membrane thickening, which initially manifests clinically as proteinuria and ultimately contributes to the development of CKD. Notably, nephrectomy alone may confer greater risk for long-term reduction in eGFR than the isolated use of nephrotoxic chemotherapy such as ifosfamide or cisplatin [28].

### Hematopoietic stem cell transplant

HSCT is the process of infusing autologous or allogenic multipotent hematopoietic cells harvested from bone marrow, peripheral blood, or umbilical cord blood to treat malignancy and other hematologic and autoimmune conditions. HSCT is preceded by a conditioning phase designed to reduce cancer burden and immunosuppress in preparation for engrafting. Conditioning regimens vary in intensity (i.e., myeloablative vs. non-myeloablative) and involve administering high-dose chemotherapy, total body irradiation, or a combination of both. While these regimens allow for improved engraftment outcomes, chemotherapy and radiotherapy can have individual and additive negative short- and long-term impacts on the kidneys. Additionally, HSCT can lead to early complications of endothelial origin that affect the kidneys, including hepatic sinusoidal obstruction syndrome (SOS, previously called veno-occlusive disease) and transplant-associated thrombotic microangiopathy (TA-TMA) (Fig. 3) [29].

#### Hepatic sinusoidal obstruction syndrome

Hepatic SOS is a life-threatening complication of HSCT. It occurs in about 30% of children receiving HSCT and is more common with allogeneic transplant and use of myeloablative conditioning, as opposed to reduced intensity regimens [30]. SOS results from chemotherapy and/or radiotherapy-induced endothelial injury, which triggers activation of the coagulation cascade. Ultimately, thrombus formation in the hepatic microvasculature leads to obstruction of hepatic sinusoids and portal hypertension. SOS generally occurs within 3 weeks of HSCT and presents with painful hepatomegaly, ascites, weight gain, and jaundice with lab evidence of hyperbilirubinemia, elevation of liver enzymes, and thrombocytopenia. Severe SOS can lead to multi-organ failure with pulmonary dysfunction, kidney injury, and encephalopathy because of portal hypertension and pro-inflammatory cytokine release. Kidney injury occurs in up to 80% of patients with SOS primarily due to hepatorenal syndrome [31]. As portal hypertension worsens, endothelial nitric oxide is released from the portal vasculature. This triggers arterial vasodilation of both the splanchnic and systemic circulation. Altogether, vasodilation results in pooling of blood in the splanchnic vasculature and decreased peripheral vascular resistance. Renal hypoperfusion and aortic baroreceptors subsequently trigger activation of compensatory pathways including the renin–angiotensin–aldosterone system, sympathetic nervous system, and antidiuretic hormone release. This leads to a maladaptive response where sodium and water retention worsen preexisting volume overload, and excessive renal vasoconstriction further promotes renal hypoperfusion and injury.

#### Transplant-associated thrombotic microangiopathy

Similarly, TA-TMA is a life-threatening complication triggered by endothelial injury that occurs in 20–30% of patients after HSCT. TA-TMA is more common after allogenic HSCT than autologous HSCT, and it is associated with the use of total body radiation, calcineurin inhibitors, and mammalian target of rapamycin inhibitors [32]. Etiology is multifactorial and likely involves interplay between the factors described in the three-hit hypothesis proposed by Dvorak et al.: an underlying predisposition to pro-inflammatory complement-mediated immune activation, treatment-induced endothelial injury, and other insults (i.e., medication toxicity, infection, or immune-mediated damage) [33]. Pathophysiology begins with activation of the coagulation cascade, complement system, and release of pro-inflammatory cytokines after endothelial cell injury. Microthrombi formation ensues and then leads to intravascular hemolysis and further endothelial damage. A systemic pro-thrombotic and pro-inflammatory response is perpetuated by intricate cross-play between complement, cytokines, and neutrophils [34, 35].

TA-TMA results in microangiopathic hemolytic anemia and therefore shares clinical features with hemolytic uremic syndrome and thrombotic thrombocytopenic purpura. It presents with anemia, thrombocytopenia, elevated lactate dehydrogenase, and schistocytes in peripheral blood. Additionally, while the kidneys are the most frequently involved organ, they are not affected in all cases of TA-TMA [36]. Manifestations of kidney involvement include reduced GFR, proteinuria, and hypertension. The disease can be mild and self-limited or lead to multi-organ failure. Severe disease is often seen in children with neuroblastoma and is more common after high-dose chemotherapy conditioning regimens [37]. Multi-organ dysfunction includes kidney failure, bowel ischemia, pulmonary hypertension, encephalopathy, and anasarca from vascular leak in the setting of generalized endothelial dysfunction [37].

Diagnosis of TA-TMA can be challenging and requires high clinical suspicion, as many of its manifestations overlap with other conditions, and its time of onset is variable, with delayed cases occurring nearly a year after HSCT. Expert consensus recommends the use of the modified Jodele Criteria for diagnosis in children, which requires the presence of ≥ 4 criteria within 2 weeks at 2 consecutive time points. Criteria include anemia, thrombocytopenia, elevated lactate dehydrogenase, presence of schistocytes, hypertension, proteinuria, and elevation of terminal complement complex sC5b-9 [36]. While histopathologic findings of TMA (i.e., glomerular fibrin deposition, narrowed and occluded capillary lumens, fragmented red blood cells, basement membrane duplication, and endothelial edema) are sufficient for diagnosis, kidney biopsy is often unnecessary due to the existence of non-invasive clinical criteria and is rarely done because of the increased risk of biopsy complications in thrombocytopenic and hypertensive patients. Kidney biopsy is indicated mainly in cases of diagnostic uncertainty [38].

Management of TA-TMA requires supportive care for end-organ dysfunction and aggressive blood pressure control. Targeted therapy is evolving and currently involves off-label use of immune-modulating drugs for complement blockade (i.e., eculizumab, a monoclonal anti-C5 antibody that inhibits formation of the terminal sC5b9 membrane attack complex; narsoplimab, a monoclonal anti-MASP-2 antibody that blocks the lectin pathway) and defibrotide for its pro-fibrinolytic, anti-thrombotic, and anti-inflammatory effects.

Despite advances in treatment, children undergoing HSCT are at particular risk for adverse kidney outcomes because of conditioning therapy and complications of HSCT. About 15–21% of children develop AKI after HSCT [39]. Some pediatric cohorts have found that kidney replacement therapy is required in up to 50% of patients who develop AKI after HSCT [40]. CKD and kidney failure are seen in up to 45% and 16% of long-term CCS with a history of HSCT, respectively [39, 41]. Notably, hemodialysis outcomes in children after HSCT are poor, and kidney failure in this survivor cohort carries a higher risk of mortality than kidney failure of other causes [42]. However, while long-term data are lacking, short-term outcomes including graft and overall survival after kidney transplant in patients with a history of HSCT are promising [43].

## Screening for chronic kidney disease in childhood cancer survivors

The acute and chronic repercussions of malignancy and its treatment on kidney health have resulted in higher short- and long-term utilization of nephrology services and the emergence of onco-nephrology [44, 45]. Approximately 20% of CCS develop chronic kidney disease from exposure to these nephrotoxic treatment modalities. Two percent of survivors progress to kidney failure (GFR < 15 ml/min/1.73 m^2^) requiring kidney replacement therapy, and less than 1% of survivors undergo kidney transplant [6, 46]. Screening strategies are therefore essential for early recognition of impaired kidney function and prompt intervention to mitigate progressive decline and the need for dialysis in CCS.

Hypertension, proteinuria, electrolyte derangements, and reduced eGFR reflect varying degrees of impaired kidney function and are associated with the risk of kidney failure. Current North American guidelines from the Children’s Oncology Group (COG) therefore recommend screening cancer survivors with a history of exposure to nephrotoxic therapies—including ifosfamide, platinum-based chemotherapy, abdominal radiation, and nephrectomy—for kidney disease with yearly blood pressure measurements and baseline lab work at the time of entry into long-term follow-up programs, with a plan to repeat as clinically indicated [47]. Children who have undergone HSCT are screened annually with blood pressure measurement, urinalysis, basic metabolic panel, and magnesium and phosphate levels, due to their particularly increased risk of CKD and kidney failure because of therapy [47]. Similarly, European guidelines suggest estimating GFR using serum creatinine and measuring urine creatinine and urine protein at least every 5 years in survivors with a history of ifosfamide, cisplatin, or carboplatin use, radiotherapy exposing the kidney or urinary tract, nephrectomy, and/or HSCT. It is also recommended that survivors treated with ifosfamide, cisplatin, or carboplatin have serum electrolytes, serum albumin, and urine glucose and phosphate measured at least every 5 years [48].

Notably, although a significant proportion of CCS have masked hypertension, current guidelines do not include ambulatory blood pressure monitoring (ABPM) as a routine screening tool. A single-center study showed that over 50% of CCS referred to nephrology had a diagnosis of hypertension based on measurements in clinic or ABPM. Close to 20% of those who completed ABPM were found to have masked hypertension [49]. Because hypertension is a modifiable cardiovascular risk factor, detection of masked hypertension, which is only possible with ABPM, could potentially improve long-term cardiovascular morbidity in CCS, although larger and longer-term studies are needed to establish optimal screening strategies. Additionally, hypertension in CCS is the strongest predictor of progression to kidney failure [5, 50]. Hypertension is present in up to 38% of CCS, and its onset within 5 years of cancer diagnosis can be associated with an eightfold risk of late kidney failure [6, 49–51].

## Predicting chronic kidney disease in childhood cancer survivors

It can be challenging to differentiate between survivors who are likely to progress to kidney failure versus those who will not. In 2023, Wu et al. published a prediction model to identify CCS at highest risk for developing kidney failure by 40 years of age [50]. This multicenter study included 5-year survivors of common childhood cancers diagnosed before 21 years of age between 1970 and 1999 (*n* = 25,483) and a sibling control group (*n* = 5045). The model was created using the Childhood Cancer Survivor Study (CCSS) cohort (*n* = 25,483) and then validated with cohorts from the Saint Jude’s Lifetime Cohort Study (SJLIFE) (*n* = 2490) and National Wilms Tumor Study (NWTS) (*n* = 396). Authors found a 1% cumulative incidence of kidney failure by age 40 years in CCS versus 0.2% in siblings. The model categorized survivors into low risk (77–80% of CCS; cumulative incidence of kidney failure 0.6%), moderate risk (17–21%; 2.3%), and high risk (2–3%; > 5%) based on their age and age at cancer diagnosis, race/ethnicity, presence of congenital urinary tract anomalies, history of nephrectomy within 5 years of diagnosis, exposure to and cumulative dosing of nephrotoxic chemotherapy, exposure to abdominal radiotherapy and cumulative radiotherapy dosing, secondary malignancy, and new-onset hypertension or diabetes within 5 years of cancer diagnosis. Notably, the incidence of kidney failure was higher in CCS than siblings regardless of risk stratification, and even survivors in the low-risk group had significantly greater probability of developing kidney failure compared to siblings (relative risk 3.2, 95% CI 1.6–6.1). Hypertension, which is a modifiable risk factor, was the most influential predictor of kidney failure. This model is available as an online clinical tool capable of identifying CCS at highest risk of kidney failure and could thus influence appropriate screening strategies, promote early detection and intervention, and potentially slow progression to kidney failure and prolong the bridge to transplant.

## Considerations prior to kidney transplant

Kidneys are the most frequently transplanted solid organ in CCS [46]. The incidence of kidney transplant in CCS is a rare but severe adverse event (0.54%) and occurs most commonly in children with a history of kidney tumors, acute lymphoblastic leukemia, and non-Hodgkin lymphoma [46]. The main risk factors associated with the need for a transplant include a history of ifosfamide use, total body irradiation, and nephrectomy [46].

Determining the appropriateness of kidney transplant in patients with a history of malignancy is complex. The American Society of Transplantation (AST) recommends considering multiple factors grouped into four key areas: organ failure factors, which weigh the risks and benefits of transplantation versus remaining on dialysis; cancer factors including duration of remission, risk of cancer recurrence, and response to therapy; immunosuppression factors such as the degree of immunosuppression needed to avoid rejection and its role in cancer recurrence; and patient factors, which include modifiable and non-modifiable risk factors and their potential impact on graft and overall survival, as well as the effect of disease and therapy on the patient’s quality of life [52]. Ultimately, the decision to proceed with transplant hinges on balancing the risk of cancer recurrence and other adverse outcomes with the potential significant benefits of transplantation, and requires shared decision-making between patients, caregivers, and their multidisciplinary team.

Similarly, deciding the timing of transplantation can be challenging. The optimal time to perform a transplant is controversial and patient-dependent. Among other things, determining the appropriate timing involves balancing the 20-fold increased cardiovascular risk associated with prolonged time on dialysis with the risk of cancer and its associated morbidity and mortality [53]. While multiple studies have not found a statistically significant association between the timing of transplant and cancer-specific mortality, this seems to be increased within the first 2 years after transplant [54, 55]. Because data reflecting outcomes associated with time from remission to transplant vary widely across studies, current guidelines offer differing evidence-based recommendations (Table 2). Notably, recommendations are made primarily for cancer types that are more common in adults. Except for certain curatively treated non-metastatic cancers, KDIGO guidelines recommend a 2–5 year wait period between remission and kidney transplant depending on cancer type and stage. Conversely, AST suggests waiting only 1–2 years to transplant if the 5-year cancer survival rate is anticipated to be at least 80%. Norwegian guidelines also recommend shorter wait times of 1 year after remission, supported by evidence which shows that kidney transplant according to this policy was not associated with decreased graft survival or decreased overall survival compared to matched controls without pre-transplant malignancy [54, 56–58]. Although transplant readiness should be assessed on a case-by-case basis, longer waiting periods as recommended for certain adult cancers may not be required in children, particularly because children generally have fewer comorbidities than adults and multiple pediatric malignancies have 5-year survival rates > 80%.

**Table 2:** Recommendations for timing of solid organ transplant after cancer remission

	KDIGO (2020)	American Society of Transplantation Consensus (2021)	Norwegian Guidelines (2015)
General time from cancer remission to solid organ transplant	2–5 years for most cancer types*	1–2 years for cancers with 5-year survival rate ~ 80%	1 year for most cancer types* Expected survival should exceed 2 years after transplant
Hematologic malignancies	• Hodgin lymphoma: localized, 2 years. Regional, 3–5 years. Distant, 5 years • Non-Hodgkin lymphoma: localized, 2 years. Regional, 3–5 years. Distant, 5 years • Post-transplant lymphoproliferative disease: nodal, 2 years. Extranodal and cerebral, 5 years • Avoid transplanting patients with leukemia or lymphoma until after receipt of curative therapy, in complete remission, and cancer free for length of time to be determined with collaboration with oncologist, patient, and transplant team	• Diffuse large B-cell lymphoma, follicular lymphoma, peripheral T cell lymphoma, Burkitt lymphoma, Hodgkin lymphoma: 2 years • Monoclonal B-cell lymphocytosis: no wait time	-
Solid tumors	• Kidney: incidentaloma (< 3 cm), no wait time. Early stage, 2 years. Large and invasive, 5 years • Sarcoma: 5 years	• Renal cell carcinoma: no wait time for Stage T1a (≤ 4 cm), N0, M0; or T1b (> 4- ≤ 7 cm), N0, M0 with follicular grade 1–2. T1b (> 4– ≤ 7 cm), N0, M0 with follicular grade 3–4, 1–2 years. T2 (7–10 cm), N0, M0, 2 years. T3, N0, M0; or T4, N0, M0, minimum of 2 years. Any T, Node +, Metastatic; Any T with sarcomatoid and/or rhabdoid histologic features; collecting duct or medullary renal cell carcinoma are not kidney transplant candidates	-

Time from remission to transplant should be assessed on a case-by-case basis considering cancer type, stage, grade, recurrence risk, and comorbidities. Cancer-specific guidelines for timing of solid organ transplant after remission are available mainly for malignancies that are common in adult populations such as breast cancer, colon cancer, prostate cancer, and lung cancer. Remission time recommendations for cancers that may be relevant in pediatric populations, if available, are specified in the table. *Guidelines do not recommend a minimum wait time between remission and kidney transplant after certain localized malignancies, including non-melanoma skin cancers, prostate cancer, and low-grade renal cell carcinoma *KDIGO* Kidney Disease Improving Global Outcomes

## Cancer risk after kidney transplant

### *De novo *cancer

Roughly 15–20% of pediatric kidney transplant recipients develop cancer by 25-year follow-up [59]. The most common new cancers to develop after pediatric kidney transplant are non-melanoma skin cell cancers (i.e., squamous cell carcinoma and basal cell carcinoma, 50%) and post-transplant lymphoproliferative disorder (PTLD, 30–35%) [59, 60]. In general, both the risk and incidence of cancer in kidney transplant recipients increase over time. Cancer incidence in adult cohorts can rise from 3 to 15% and 55% at 40, 60, and 80 years of age, respectively, in those with long-term graft survival > 20 years, and age itself is considered a risk factor [61]. In pediatric cohorts, the cumulative incidence of cancer has been reported to be approximately 2%, 4%, 7%, and 10% at 1, 5, 10, and 15 years after transplant, respectively [62]. Additionally, older age at the time of kidney transplant has been shown in other cohorts to influence risk, as those transplanted during adolescence developed post-transplant malignancy more frequently than young transplant recipients under the age of 3 years [60].

There are few studies on risk factors, incidence, and mortality of cancer recurrence or de novo cancer in CCS after kidney transplant specifically. A cohort study utilizing registry and administrative data from Canada reported that the 10-year cumulative incidence of de novo cancer in 52 CCS receiving solid organ transplants (SOTs) was 8.6%, which was not statistically significantly different than the incidence of first cancer in SOT recipients without a history of malignancy [63]. A large adult population-based cohort study showed that approximately 10% of kidney transplant recipients with a history of malignancy developed cancer recurrence and 10% developed de novo cancer at a median of 1.5 and 2 years after transplant, respectively [64]. Additionally, de novo malignancies observed in cancer survivors after kidney transplant often mirror those seen in cohorts without a history of cancer, with PTLD and non-melanoma skin cancers being the most common [65]. Although these previously mentioned studies are quite informative, variability in population and cancer type makes it challenging for findings to be generalized to CCS.

### Cancer recurrence

CCS kidney transplant recipients are at risk for developing cancer recurrence because of the immunosuppression used to prevent graft rejection. Rates and timing of recurrence vary widely by cancer type and other factors, such as patient age, staging at diagnosis, underlying cytogenetics, and time from cancer remission to organ transplant. In general, cancer recurrence is most common within the first 5 years of remission, and late recurrence (> 5 years after remission) occurs less frequently. In a meta-analysis assessing cancer recurrence after SOT in adults, Acuna et al. found that transplant < 5 years from cancer diagnosis was associated with a 2.8 times higher risk of primary cancer recurrence compared to waiting 5 years or more. Additionally, primary cancer recurrence rates were higher in adult recipients after kidney transplant (2.4 events per 100 person-years) than after other SOT [66]. In CCS overall, cumulative incidence of late recurrence is approximately 4.4%, 5.6%, and 6.2% at 10, 15, and 20 years, respectively, and varies by cancer diagnosis [67].

### Post-transplant lymphoproliferative disorder

PTLD represents a spectrum of disease characterized by benign polyclonal to malignant monoclonal B-cell (95%), T-cell, or T/NK cell proliferation in the setting of immunosuppression after HSCT or SOT [68]. It develops in up to 4% and 20% of HSCT and SOT recipients, respectively, and is less common after kidney or liver transplant (1–5%) than other SOT such as heart and lung (2–10%) and intestinal or multi-visceral transplants (5–20%), perhaps due to the lower degree of immunosuppression required to prevent graft rejection [69, 70]. The onset of PTLD after SOT has a bimodal distribution; most cases occur within the first 1–2 years, while the second peak in incidence is seen around the 5- to 10-year mark [71]. The risk for PTLD increases with a history of prior malignancy, a higher degree of immunosuppression (i.e., use of anti-thymoglobulin), and Epstein-Barr virus (EBV) infection—particularly if acquired after transplant [72, 73].

Pathogenesis of PTLD can be EBV-mediated (90%) or non-EBV-mediated [74]. Transplant immunosuppression interferes with both CD8 + and CD4 + T-cell surveillance. After EBV infection, T-cells therefore fail to respond to viral antigen presentation. This allows activation of latent oncogenic viral proteins that drive B-cell proliferation and inhibit tumor suppressor genes, resulting in longer cellular lifespan and acquisition of genetic mutations that ultimately lead to EBV-mediated PTLD [70]. Non-EBV-mediated disease is often associated with T-cell lymphoproliferation and accounts for nearly half of PTLD cases after kidney transplant, but its pathogenesis is not as well understood [68, 75]

After kidney transplant, the clinical presentation of PTLD may vary from asymptomatic to life-threatening. Often, patients present with non-specific B symptoms, lymph node enlargement, hepatosplenomegaly, cytopenia, and/or tumor lysis syndrome in the setting of lymphoproliferation. Twenty to 25% of patients have direct infiltration of the grafted kidney resulting in graft dysfunction. Patients may also present with symptoms related to the infiltration of other areas such as the abdomen (60–70%), chest (45–65%), head and neck (20–30%), and central nervous system (20–25%) [74]. Clinical suspicion of PTLD can be seen on PET scan and ultimately confirmed with tissue biopsy. Treatment and prevention are centered around reducing immunosuppression, but antivirals, immunomodulators, and chemotherapy are often needed. PTLD and its treatment are therefore associated with significant risk for allograft dysfunction and up to a 50% 5-year mortality rate [71].

## Cancer screening in kidney transplant recipients

While kidney transplant in CCS can be lifesaving, cancer recurrence and de novo malignancy are serious concerns because they significantly increase morbidity (including graft failure) and mortality. However, no post-transplant cancer surveillance guidelines are specifically designed for CCS. Children are therefore followed using KDIGO guidelines, which suggest monitoring immunosuppressive drug levels, allograft function, and screening for skin cancer and viral infections, including EBV.

The timing and frequency of drug level and allograft monitoring vary based on time from transplant. Due to the risk of skin cancer, guidelines recommend an annual skin and lip examination and suggest that patients with a history of skin or lip cancer or precancerous lesions be followed by a healthcare professional with experience in diagnosing and treating skin cancer. Other cancer screening plans should be made on an individual basis, but overall, patients should follow local guidelines for screening cervical, breast, prostate, and colon cancer.

EBV is monitored closely with measurement of viral load once in the first week after transplant, then monthly for the first 3–6 months, and every 3 months until the end of the first post-transplant year. Although these screening recommendations are commonly applied, quantitative EBV PCR results may be difficult to interpret. EBV viremia is common in children after kidney transplant, as it is estimated to occur in 20–60%, and viral load measurements can vary widely across blood compartments, making it difficult to establish levels that are associated with higher risk of PTLD.

Zaffiri et al. developed a screening algorithm in which patients were stratified by PTLD risk and screened for EBV accordingly [71]. Patients with high risk for PTLD included pediatric ages and the elderly, recipients with EBV seromismatch, and exposure to belatacept or anti-thymoglobulin. The authors proposed screening this subset of patients for EBV viremia monthly for the first year after transplant while keeping the KDIGO recommendations for lower-risk patients (1, 3, 6, 9, and 12 months).

## Kidney transplant outcomes in childhood cancer survivors

After SOT, cancer incidence and cancer-related mortality are significantly higher in both children and adults with a pre-transplant cancer diagnosis than in those without [54, 55, 60]. Adult data suggest that recurrence of malignancy accounts for up to 12% of deaths after kidney transplantation [76]. There are few studies that report the long-term outcomes for CCS specifically after kidney transplant. Five-year mortality rates after kidney transplant in CCS range from approximately 2 to 20% and are greatly impacted by the diagnosis of new or recurring cancer [46]. Although this is a growing area of research, current data have revealed notable differences in post-transplant outcomes between pediatric and adult cancer survivors. Adult cancer survivors who undergo kidney transplantation after a cancer diagnosis have an increased risk of cancer-specific and all-cause mortality [54, 64]. In contrast, post-transplant all-cause mortality in CCS appears like that of kidney transplant recipients without a history of malignancy, despite a higher risk of cancer-related mortality [77, 78]. However, it is important to highlight that post-transplant malignancy significantly contributes to mortality in pediatric kidney transplant recipients regardless of pre-transplant malignancy, as roughly 10–20% of deaths after transplant are linked to a new cancer diagnosis in children without a history of prior malignancy [60, 79].

Importantly, there does not appear to be a significant difference in graft survival or overall mortality 25 years after transplant based on survival analysis of CCS patients with pre-transplant malignancy versus those without [77]. Additionally, kidney transplant in CCS has the highest 5-year survival rate (93.5%) of other SOT, including heart (80%), liver (28%), and lung (34%) [46]. Altogether, these promising outcomes suggest that kidney transplantation is a viable and life-extending treatment option that should be followed by thoughtful screening to mitigate the risk of new or recurrent cancer diagnosis.

## Knowledge gaps

**Fig. 4:**
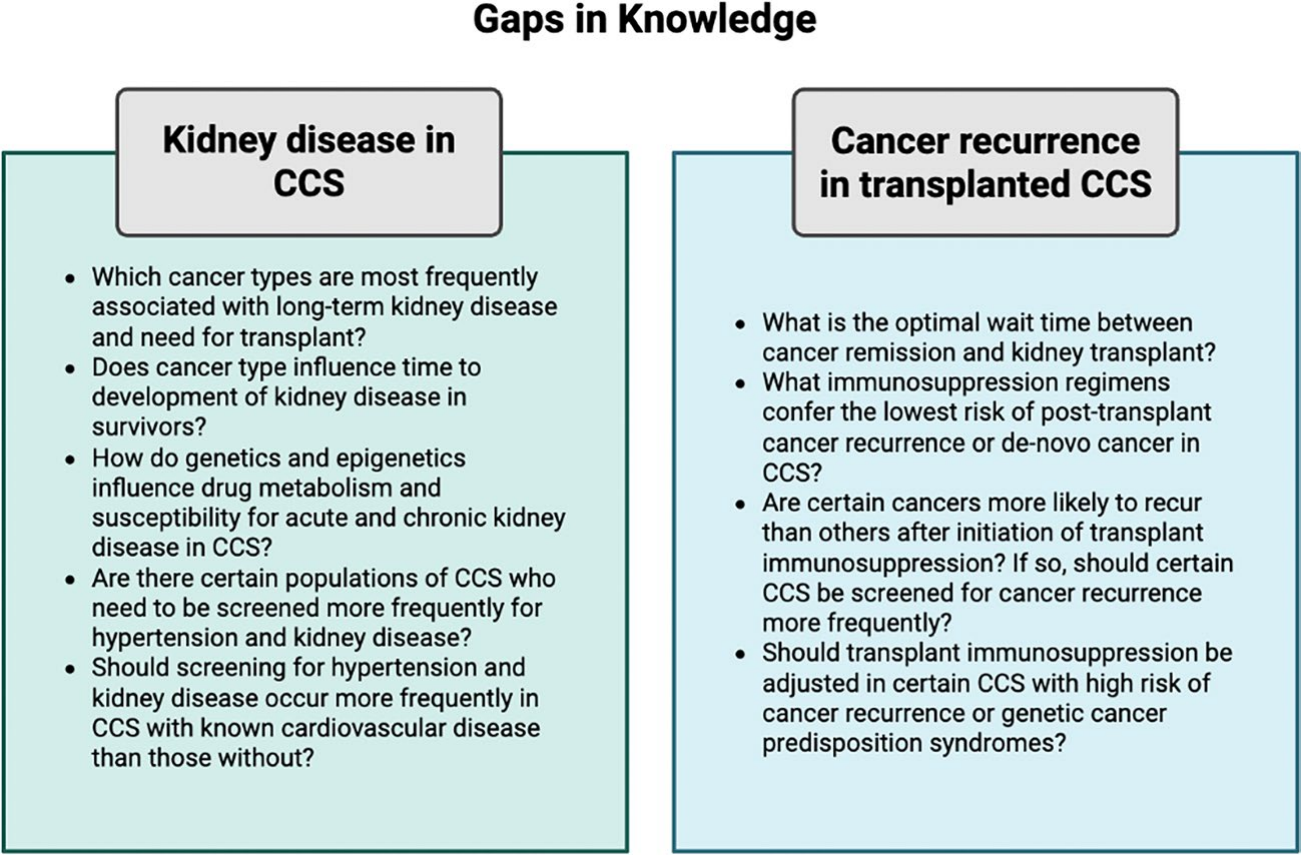
Gaps in knowledge. Abbreviations: CCS, childhood cancer survivors. Created in BioRender. Kane A (2025) https://BioRender.com/82t42wp

Overall, much research is still needed to understand the implications of a prior cancer diagnosis on kidney transplant outcomes. As cancer treatments improve and the CCS population and life expectancy increase, there will likely be a continued rise in the prevalence of kidney disease in survivors and thus a possible growing need for kidney transplant. Understanding the short- and long-term complications and survival outcomes in CCS transplant recipients and how they differ from recipients without a history of malignancy is therefore crucial, as this will help tailor existing adult recommendations for the timing of kidney transplant after a cancer diagnosis and screening recommendations for post-transplant comorbidities, including de novo cancer and cancer recurrence, in children. Figure 4 depicts multiple questions that warrant further exploration, highlighting key areas for continued research.

## Conclusions

Advances in pediatric cancer therapy have improved survival rates and led to a growing population of CCS who face long-term health challenges associated with adverse effects of cancer treatment, including kidney dysfunction. Current kidney disease screening strategies can be further optimized using clinically available patient-centered risk prediction models to identify survivors with the highest probability of developing kidney failure. While the need for kidney transplant is overall rare in CCS, kidneys are the most frequently transplanted solid organ in CCS, and further research is needed to achieve a pediatric consensus for the ideal timing of transplant after cancer diagnosis, which remains controversial. Although cancer recurrence and de novo malignancy in the setting of transplant immunosuppression pose a significant burden on morbidity and mortality for survivors, cancer history prior to transplant does not appear to negatively impact long-term graft function and overall survival in childhood cancer survivors based on current published data. Overall, further research is needed to refine kidney disease screening strategies in high-risk CCS and to optimize transplant timing and care to reduce post-transplant cancer-related morbidity and mortality in this unique growing pediatric population.

## Key summary points

1. Cancer survival rates have markedly improved with modern treatment protocols, but cancer therapy can result in early and late adverse kidney effects. Approximately 20% of CCS develop CKD, 2% progress to kidney failure and < 1% undergo kidney transplant.
2. In CCS, hypertension is the strongest predictor of progression to kidney failure and may present as masked hypertension evident only on ABPM, which is not currently included in routine CCS screening guidelines.
3. There is no pediatric-specific consensus for determining the optimal time from cancer remission to kidney transplant. Similarly, there are no specific cancer screening guidelines for CCS after kidney transplant.
4. Post-transplant cancer incidence and cancer-related mortality are higher in children with history of malignancy prior to transplant than in those without, but graft survival and overall mortality seem to be similar between both groups.

## Multiple choice questions

Answers appear following References.

1. Which of the following statements is true regarding a CCS’s risk for kidney disease?
  - A childhood cancer survivor’s risk for CKD and kidney failure is comparable to that of the general population
  - CCS at highest risk of kidney disease include those with history of HSCT, TBI, nephrectomy or use of nephrotoxic chemotherapy regimens
  - CCS at lowest risk of kidney disease include those with history of unilateral nephrectomy receiving chemotherapy regimens that contain cyclophosphamide and carboplatin
  - The risk of developing radiation nephropathy is highest with total radiotherapy doses under 15 Gy
2. Which of the following statements is true regarding the incidence of CKD and kidney failure in CCS?
  - Approximately 50% of CCS develop CKD
  - Approximately 15% of CCS who develop CKD progress to kidney failure
  - Approximately 20% of CCS develop CKD
  - Approximately 5% of CCS with CKD progress to kidney failure
  - Both B and C are correct
3. What are the three most reported de novo cancer types to develop after KT in children?
  - Melanoma, PTLD and cervical cancer
  - Basal cell carcinoma, squamous cell carcinoma and PTLD
  - PTLD, basal cell carcinoma and renal cell carcinoma
  - Kaposi sarcoma, basal cell carcinoma and PTLD
4. Which of the following statements is true regarding transplant wait times in adults?
  - While shorter waiting times from cancer remission to time of transplant may be associated with increased risk of cancer recurrence, this does not appear to affect graft survival or overall mortality in adults
  - Transplantation within a year of cancer remission is associated with decreased risk for cancer recurrence
  - One- to two-year transplant wait times are recommended by the American Society of Transplantation when 5-year cancer survival rates approach 50%.
  - KDIGO guidelines recommend a 5–7 year wait period between remission and kidney transplant, regardless of cancer type
5. Which of the following is NOT a risk factor for PTLD?
  - History of prior malignancy
  - Transplant recipient that is EBV-negative with a donor that is EBV-positive
  - Transplant recipient that is EBV-negative who acquires EBV infection post-transplant
  - Transplant recipient that is EBV-negative with a donor that is EBV-negative

## Supplementary Information

Below is the link to the electronic supplementary material.

Graphical abstract (PPTX 1.34 MB)
