# Supplementary material for: Kidney disease and transplantation in childhood cancer survivors
Source: Pediatr Nephrol. 2025 Oct 20;41(7):1967–81. doi: 10.1007/s00467-025-06985-x (PMC13197264; doi:10.1007/s00467-025-06985-x)
Supplement: Supplementary file 1 — Graphical abstract (PPTX 1.34 MB) [file 467_2025_6985_MOESM1_ESM.pptx]

## Slide 1
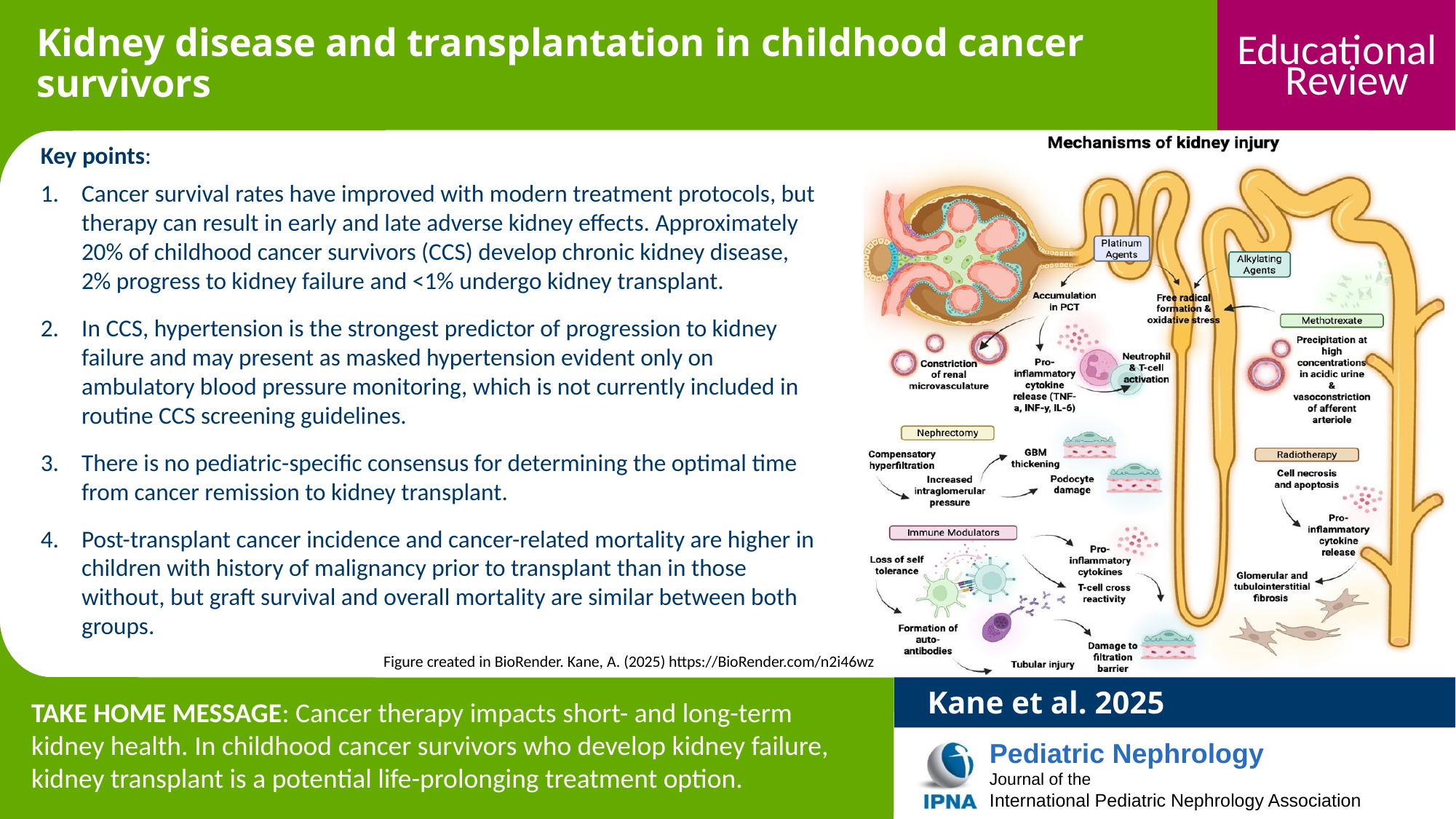

Kidney disease and transplantation in childhood cancer survivors
Key points:
Cancer survival rates have improved with modern treatment protocols, but therapy can result in early and late adverse kidney effects. Approximately 20% of childhood cancer survivors (CCS) develop chronic kidney disease, 2% progress to kidney failure and <1% undergo kidney transplant.
In CCS, hypertension is the strongest predictor of progression to kidney failure and may present as masked hypertension evident only on ambulatory blood pressure monitoring, which is not currently included in routine CCS screening guidelines.
There is no pediatric-specific consensus for determining the optimal time from cancer remission to kidney transplant.
Post-transplant cancer incidence and cancer-related mortality are higher in children with history of malignancy prior to transplant than in those without, but graft survival and overall mortality are similar between both groups.
Figure created in BioRender. Kane, A. (2025) https://BioRender.com/n2i46wz
Kane et al. 2025
TAKE HOME MESSAGE: Cancer therapy impacts short- and long-term kidney health. In childhood cancer survivors who develop kidney failure, kidney transplant is a potential life-prolonging treatment option.
